# Effects of litter and root manipulations on soil carbon and nitrogen in a Schrenk’s spruce (*Picea schrenkiana*) forest

**DOI:** 10.1371/journal.pone.0247725

**Published:** 2021-02-25

**Authors:** Haiqiang Zhu, Lu Gong, Zhaolong Ding, Yuefeng Li

**Affiliations:** 1 College of Resources and Environment Science, Xinjiang University, Urumqi, China; 2 Ministry of Education, Key Laboratory of Oasis Ecology, Urumqi, China; Fujian Normal University, CHINA

## Abstract

Plant detritus represents the major source of soil carbon (C) and nitrogen (N), and changes in its quantity can influence below-ground biogeochemical processes in forests. However, we lack a mechanistic understanding of how above- and belowground detrital inputs affect soil C and N in mountain forests in an arid land. Here, we explored the effects of litter and root manipulations (control (CK), doubled litter input (DL), removal of litter (NL), root exclusion (NR), and a combination of litter removal and root exclusion (NI)) on soil C and N concentrations, enzyme activity and microbial biomass during a 2-year field experiment. We found that DL had no significant effect on soil total organic carbon (SOC) and total nitrogen (TN) but significantly increased soil dissolved organic carbon (DOC), microbial biomass C, N and inorganic N as well as soil cellulase, phosphatase and peroxidase activities. Conversely, NL and NR reduced soil C and N concentrations and enzyme activities. We also found an increase in the biomass of soil bacteria, fungi and actinomycetes in the DL treatment, while NL reduced the biomass of gram-positive bacteria, gram-negative bacteria and fungi by 5.15%, 17.50% and 14.17%, respectively. The NR decreased the biomass of these three taxonomic groups by 8.97%, 22.11% and 21.36%, respectively. Correlation analysis showed that soil biotic factors (enzyme activity and microbial biomass) and abiotic factors (soil moisture content) significantly controlled the change in soil C and N concentrations (*P* < 0.01). In brief, we found that the short-term input of plant detritus could markedly affect the concentrations and biological characteristics of the C and N fractions in soil. The removal experiment indicated that the contribution of roots to soil nutrients is greater than that of the litter.

## Introduction

Forest soil has attracted much attention because of its enormous capacity for storing carbon (C) and nitrogen (N) [1]. Slight variations in forest soil C and N storage profoundly affect the C and N balance in terrestrial ecosystems [2]. In forest ecosystems, litter and roots are important links between plants and soil; they are primary sources of soil C and N [3] and affect the formation and turnover of soil C and N by altering soil microbial activity and the soil microenvironment (e.g. soil temperature, soil moisture and soil pH) [4, 5]. Thus, litter and root turnover play a critical role in the C and N cycles between plants and soil [4]. However, environmental change (e.g. global warming, increased precipitation, drought) will significantly impact the net primary productivity of forests, thereby changing the quantity of aboveground and belowground litter input into the soil [6]. Changes in the quantity of aboveground and belowground litter can change soil physicochemical properties [7], and soil microbial communities [8] and ultimately have an important impact on biogeochemical processes [4].

In situ manipulation experiments, such as detritus input and removal treatment (DIRT), can explore the effect of plant detritus on soil C and N characteristics by changing the amount of roots and litter inputs to the soil [9, 10]. Extensive studies have also indicated that soil C and N contents have different responses to litter addition (e.g. increasing or no significant change) [11, 12]. For example, a doubled litter input (DL) treatment increased the soil organic carbon (SOC) content by 6–12% in a temperate oak forest [13]. However, in beech and poplar forests, the same treatment had no remarkable influence on SOC and total nitrogen (TN) over the 10- or 14-year study period [11, 14]. These results may be in part attributable to differences in the duration of the soil priming effect and the balance between new C/N inputs and C/N losses caused by soil mineralization [15]. The response of the soil C and N fractions to litter addition was similar to that of SOC and TN. For example, Miao et al. (2019) [16] demonstrated that soil microbial biomass carbon (MBC) and microbial biomass nitrogen (MBN) increased significantly in the DL treatment after the a 2.5-year study period. However, Wang et al. (2019) [17] found that short-term litter addition had no significant influence on soil labile C and N contents in eucalyptus forests. Furthermore, a meta-analysis based on 68 plant detritus addition experiments showed that litter removal (NL) could reduce soil C and N contents [7]. Reynolds et al. (2018) [18] reported that 20 years of NL reduced the soil C content by approximately 30% in temperate forests. However, there was no significant effect of long-term NL on MBC and MBN in hardwood forests [19]. These studies have indicated that the changing plant detritus input results in a nonlinear relationship with soil C and N [20, 21], which may be attributed to varying input characteristics and decomposition rates in different forest ecosystems, as well as soil C/N saturation and storage potential [22]. Therefore, conducting the DIRT experiment at more sites will be instrumental to comprehensively understand the effects of the changing litter and roots on the soil C and N contents of forest ecosystems.

Many studies have shown that soil C and N mainly originate from the decomposition of aboveground litter and the secretion of underground roots [23, 24]. Moreover, litter and plant roots have different material structures and decomposition rates. Therefore, scholars quantified the relative contribution of aboveground and underground detritus to soil C and N [25] and found that they have different degrees of influence on soil C and N characteristics in diverse forest ecosystems. For example, Wu et al. (2018) [26] found that the decrease in underground detritus had a greater impact on SOC and MBC contents than aboveground litter. However, in the Harvard forest, the litter removal treatment resulted in a larger decrease in soil C and N contents than that of the no-roots treatments [15]. These results may be due to changes in soil C and N budgets and factors influencing them. Additionally, previous research has mainly focused on the effect of litter and root manipulations on topsoil (0–20 cm), neglecting the changes in C and N in deeper soil and limiting the understanding of C and N turnover and determinants in deeper soil [27, 28]. Recent evidence has shown that the amount of SOC stored in deeper soil is estimated to be approximately 77% of the SOC pool [29], which functions as a potential C sink. As a crucial component of the terrestrial C cycle, the deeper soil C content is affected by environmental changes (e.g. the variation in soil water and heat factors caused by the difference in litter quantity) [30, 31]. Understanding the changes in the deeper soil C pool is critical to accurately assess the role of forest soil C in the regional C cycle. In previous studies, the impact of litter and root manipulations on soil C and N appeared context-dependent, varying across forest ecosystem types and with the duration of the experiments and litter quantity and quality [16, 17, 20]. As a result, there is uncertainty in our understanding of how forest soil C and N can respond to environmental change. Therefore, it is urgent to thoroughly study the characteristics and mechanisms of C and N cycling in different types of forest soils [11].

Soil microorganisms are important factors connecting the plant and soil material cycles and play a vital role in forest litter decomposition and the soil C and N cycles [32]. Soil microorganisms can not only transform plant-derived organic matter into soil organic matter by participating in litter decomposition and their own metabolic activities [33], but also affect the process of soil C and N degradation [34]. Previous studies indicated that increased microbial activity can accelerate the decomposition of soil C and N [35, 36]. In addition, changes in plant detritus can affect soil microbial activity and community structure by altering soil nutrient availability and stability [37]. In turn, the microbial activities control the nutrient availability to plants [38]. It is essential to consider the role of soil microorganisms to better understand the mechanism of plant detritus change on soil C, N and nutrient cycles.

Tianshan Mountain is the largest mountain forest distribution area in Xinjiang and is extremely sensitive to climate change [39]. Schrenk’s spruce is the dominant species in the Tianshan forest ecosystem and plays a vital role in fixing nitrogen, releasing oxygen, regulating the climate, and maintaining the ecological environment. Schrenk’s spruce forest systems have low-quality (high C/N ratio) litter and a shallow the root system [40]. However, the influence of this type of litter input and shallow roots on soil C and N characteristics and its mechanisms remain unclear. Therefore, we conducted a two-year experiment to investigate how root and litter inputs affect soil C and N dynamics, documenting the role of biotic and abiotic variables linking litter and roots to soil C and N. We addressed the following questions: (1) Does the short-term addition and removal of low-quality (high C/N ratio) litter and shallow roots significantly affect soil C and N pools? (2) What is the relative importance of litter and roots on soil C and N?

## Materials and methods

### Study site

The study was established in a area of Schrenk’s spruce (*Picea schrenkiana*) forest near the Nanshan Observation Station of Xinjiang Observatory, Northwest China (87.18°E, 43.47°N), at an altitude of approximately 2080 m. The region has an arid, temperate continental climate with distinct cold and warm seasons. The average annual temperature is 0–4°C, the average annual precipitation is approximately 500 mm, and the frost-free period is 88.6 days [41]. Schrenk’s spruce is a dominant species of forest ecosystem in Tianshan. The stand is mostly a pristine forest, with a height of approximately 16 m and a canopy density of 0.6–0.8. The soil of this area is mainly gray-brown forest soil over calcium rock parent material [42], which is weakly acidic and has a thick humus layer.

### Experimental design and soil sampling

**Fig 1:**
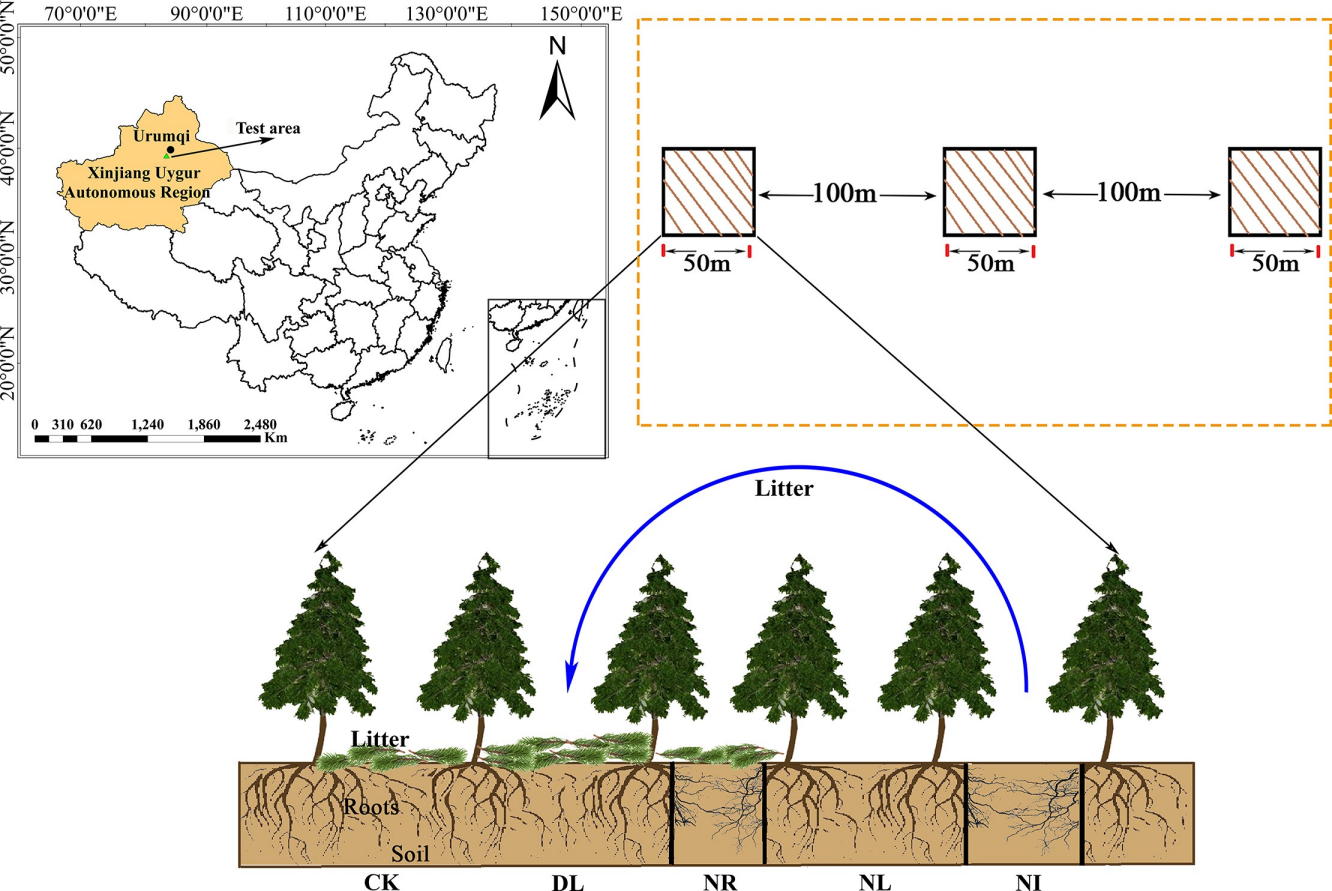
Location of this study and experiment treatments. Abbreviations refer to each treatment as follows: CK: control; DL: doubled litter inputs treatment; NL: removal of litter treatment; NR: root removal treatment; NI: root and litter exclusion treatment. black bars represent PVC boards for excluding roots.

In September 2017, three 50 m × 50 m representative plots with the same altitude, similar tree age and slope were established in the study site, with at least 100 m spacing between each plot. Five 1 m × 1 m subplots were set in each plot for different treatments: 1) the control group (CK), 2) doubled litter inputs (DL), 3) removal of litter (NL), 4) root exclusion (NR), and 5) a combination of litter removal and root exclusion (NI) (as shown in Fig 1). In the CK treatment, natural above- and belowground litter inputs were allowed. The input of aboveground litter was doubled in the double litter subplots by placing litter collected monthly from NL subplots. In the NL treatment, the aboveground litter was collected with a 100 mesh nylon net placed 0.5 m above the ground, and the litter was removed from the subplot every month. For the NR treatment, a 0.1 m wide and 1 m deep trench was dug around each subplot, and then the living roots were cut. PVC boards were inserted into the trenches to prevent new roots from growing into the subplot. Both the aboveground litter and the roots were excluded from the NI treatment.

In September 2019, soil samples were collected with a soil drill from four depths (0–10 cm, 10–20 cm, 20–30 cm, and 30–50 cm) in each 1 m × 1 m subplot. Then, residual material (e.g. plant roots and stones) was removed, and the soil was transported to the laboratory in plastic bags. Some part of the samples were stored in an ultralow temperature freezer at -80°C to determine the microbial community activity and composition. The remaining of soil samples were dried and then sieved in order to determine physical and chemical properties.

### Soil analysis

Soil C indices include SOC, dissolved organic carbon (DOC), and MBC. The SOC was measured using the potassium dichromate method [43]; DOC was determined by cold water extraction [21]; and MBC was assayed by chloroform fumigation extraction [44].

Soil N indices include TN, MBN, ammonium N and nitrate N. TN was measured by the semimicro Kjeldahl method [43]; nitrate N was determined by KCl extraction; ammonium N was measured using indophenol blue colorimetry [45]; and MBN was determined by chloroform fumigation-extraction [44].

Soil enzyme activity indices include cellulase, peroxidase, β-n-acetylglucosaminidase, and phosphatase. Cellulase activity was determined by 3,5-dinitrosalicylic acid colorimetry [46], peroxidase activity was assayed by colorimetry [47], and phosphatase activity was determined using disodium phenylphosphate colorimetry [48]. β-N-acetylglucosidase activity was analyzed with a multifunctional microplate reader [47].

Soil microbial indices include bacterial biomass, fungal biomass and actinomycete biomass. Soil microbial biomass and community composition were calculated based on phospholipid fatty acids (PLFAs). The PLFAs i14:0, a16:0, i15:0, a15:0, i16:0, i17:0 and a17:0 were used as indicators of gram-positive bacteria. The PLFAs 16:1w7c, cy17:0, 17:1w8c, 10Me17:1w7c, and 18:1w7c were used as indicators of gram-negative bacteria. The unsaturated PLFAs 18:1ω9c, 18:2ω6, 9c were used as indicators of fungi. PLFAs 10Me16:0 and 10Me17:0 were used as indicators of actinomycete [26].

### Statistical analysis

Statistical analyses were conducted using SPSS 17.0 (SPSS, IBM, USA). One-way ANOVA was used to test the effects of litter and root manipulations on soil C and N and biological characteristics, and the least significant difference (LSD) was processed to test the differences between treatments and soil layers. Redundancy analyses (RDA) were applied to identify the biotic and abiotic factors affecting soil C and N characteristics. Origin 9.0 (Origin Lab, Massachusetts, USA) and Canoco 4.5 (Biometris, Wageningen, The Netherlands) were used for drawing graphics.

## Results

### Variations in soil C concentration

**Fig 2:**
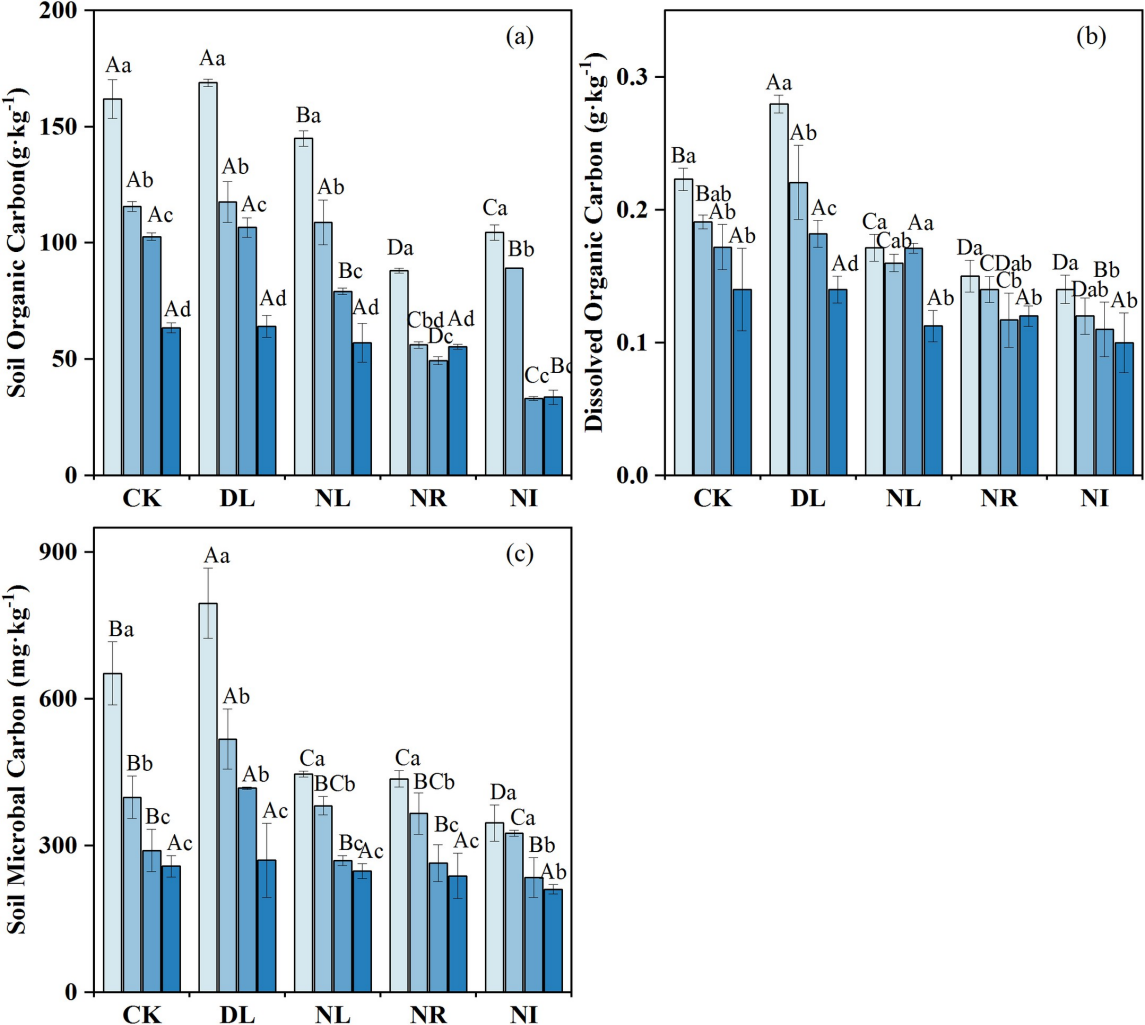
Mean soil organic carbon concentration (a), dissolved organic carbon concentration (b), and soil microbial carbon concentration (c) under different treatments (n = 12 for each treatment). Abbreviations refer to each treatment as follows: CK: control; DL: doubled litter inputs treatment; NL: removal of litter treatment; NR: root removal treatment; NI: root and litter exclusion treatment. Lowercase letters indicate differences between soil layers in the same treatment at the *p* < 0.05 level; uppercase letters indicate differences between treatments within the same soil layer at the *p* < 0.05 level.

The concentrations of SOC, DOC and MBC decreased with soil depth (Fig 2), with the concentration between 0 and 10 cm being significantly higher than that between 20 and 50 cm (*P* < 0.05). The effects of litter and root manipulations on the SOC, DOC and MBC were different (Fig 2). DL treatment had no marked effect on SOC concentration (*P* = 0.853) (Fig 2A) but had a noticeable influence on DOC (*P =* 0.039) (Fig 2B). Compared with CK, DL increased soil DOC and MBC by 17.65% and 10.89%, respectively. The NR, NL, and NI treatments decreased the soil DOC by 31.23%, 15.44%, and 35.06%, respectively, and the soil MBC by 12.88%, 7.55%, and 13.46%, respectively. In contrast, the decrease in SOC concentration in the NR treatment (-43.92%) was greater than that in the NL treatment (-12.13%).

### Variations in soil N concentration

**Fig 3:**
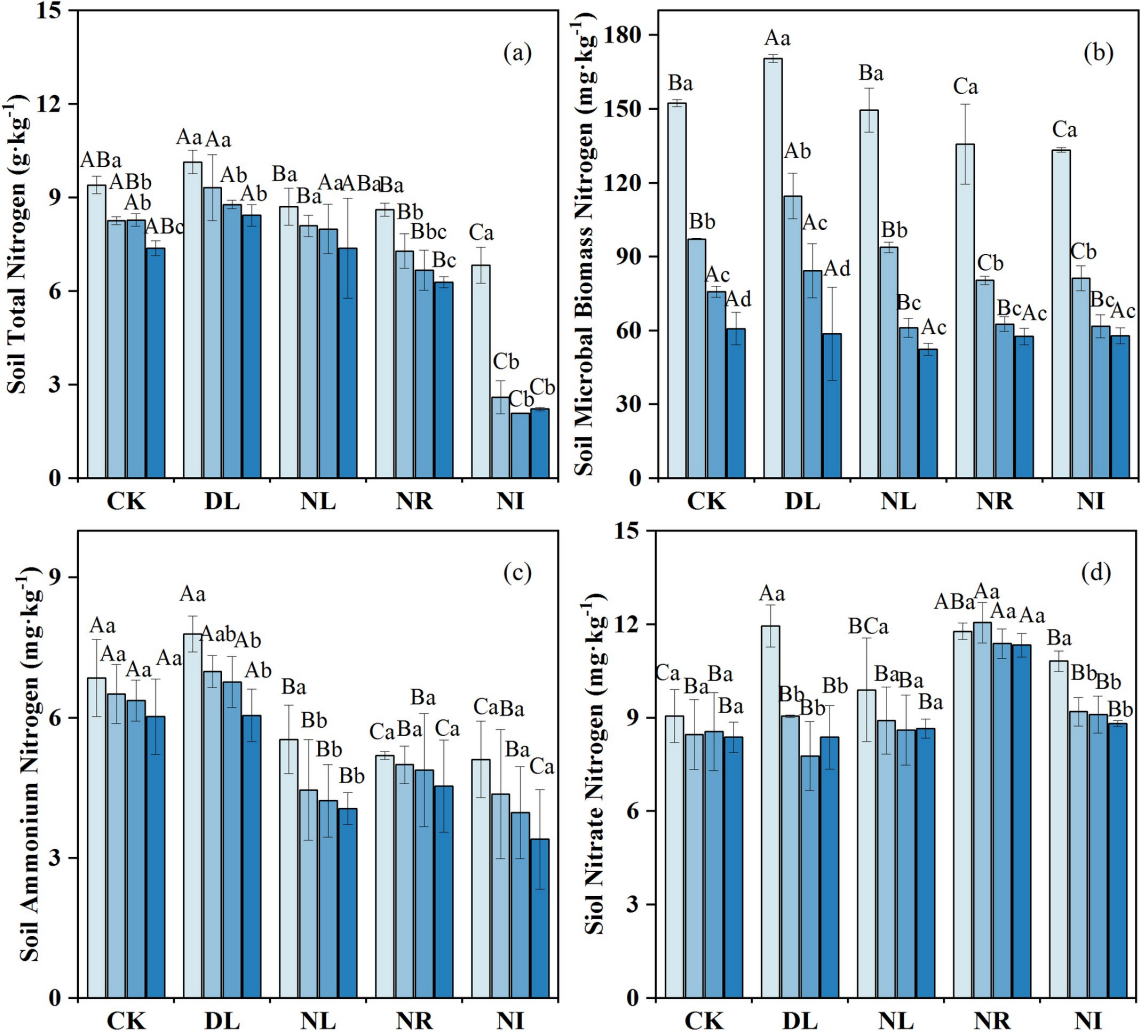
Change in soil nitrogen concentration under different treatments (n = 12 for each treatment). CK: control; DL: doubled litter inputs treatment; NL: removal of litter treatment; NR: root removal treatment; NI: root and litter exclusion treatment. Lowercase letters indicate differences between soil layers in the same treatment at the *p* < 0.05 level; uppercase letters indicate differences between treatments within the same soil layer at the *p* < 0.05 level.

The effects of litter and root manipulations on the soil TN, MBN, ammonium N and nitrate N were not consistent (Fig 3). DL did not significantly influence soil TN (*P* = 0.098) (Fig 3A) but significantly increased MBN, ammonium N, and nitrate N, which increased by 10.89%, 7.19%, and 7.79%, respectively. Compared to the CK, NL, NR and NI treatments decreased the soil TN by 3.42%, 23.48% and 55.40%, the MBN by 7.55%, 12.88% and 13.46%, and the ammonium N by 28.98%, 23.79% and 34.53%, respectively. However, the concentration of nitrate N increased by 4.69%, 35.04% and 10.07% in the NL, NR and NI treatments, respectively, compared to the CK.

### Variations in soil enzyme activity and microbial biomass

**Fig 4:**
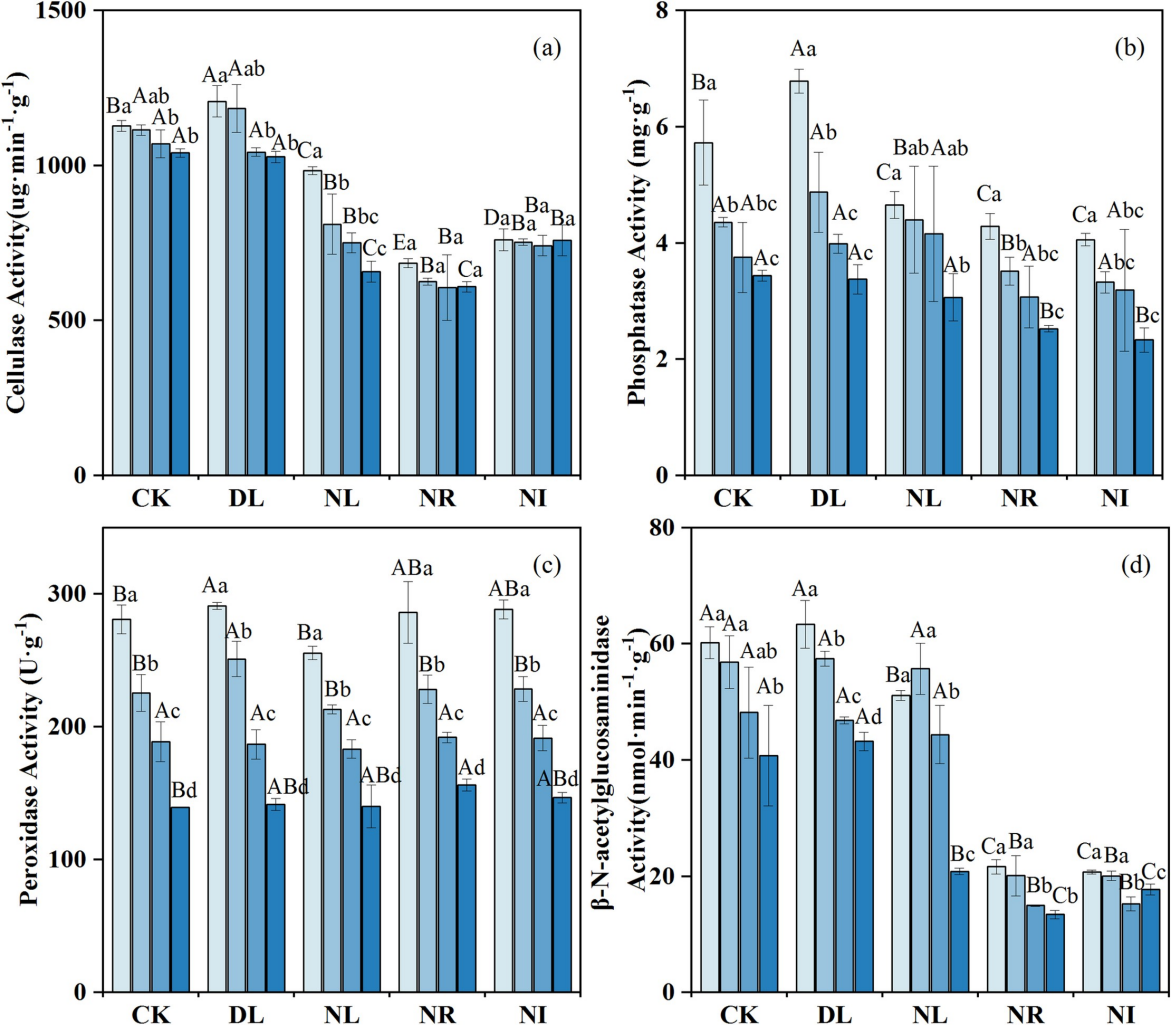
Changes in soil enzyme activities under different treatments (n = 12 for each treatment). CK: control; DL: doubled litter inputs treatment; NL: removal of litter treatment; NR: root exclusion treatment; NI: root exclusion and litter removal treatment. Lowercase letters indicate differences between soil layers in the same treatment at the *p* < 0.05 level; uppercase letters indicate differences between treatments within the same soil layer at the p < 0.05 level.

After two years of experimental manipulations, the activities of cellulase and phosphatase in the 0–20 cm layer were markedly higher in the DL treatment than in relative to the CK treatment (*P =* 0.044 and 0.047 for cellulase and phosphatase, respectively), whereas the β- N-acetylglucosaminidase activities did not significantly increase (Fig 4). The activities of cellulase, β-N-acetylglucosidase and phosphatase decreased significantly in the NR and NI treatments (NR: *P =* 0.001, *P =* 0.001, *P =* 0.015; NI: *P =* 0.001, *P =* 0.001, *P =* 0.009), but the peroxidase activities increased slightly. Moreover, litter removal and addition had a greater effect on the enzymatic activity of surface soil than in deeper soil.

**Fig 5:**
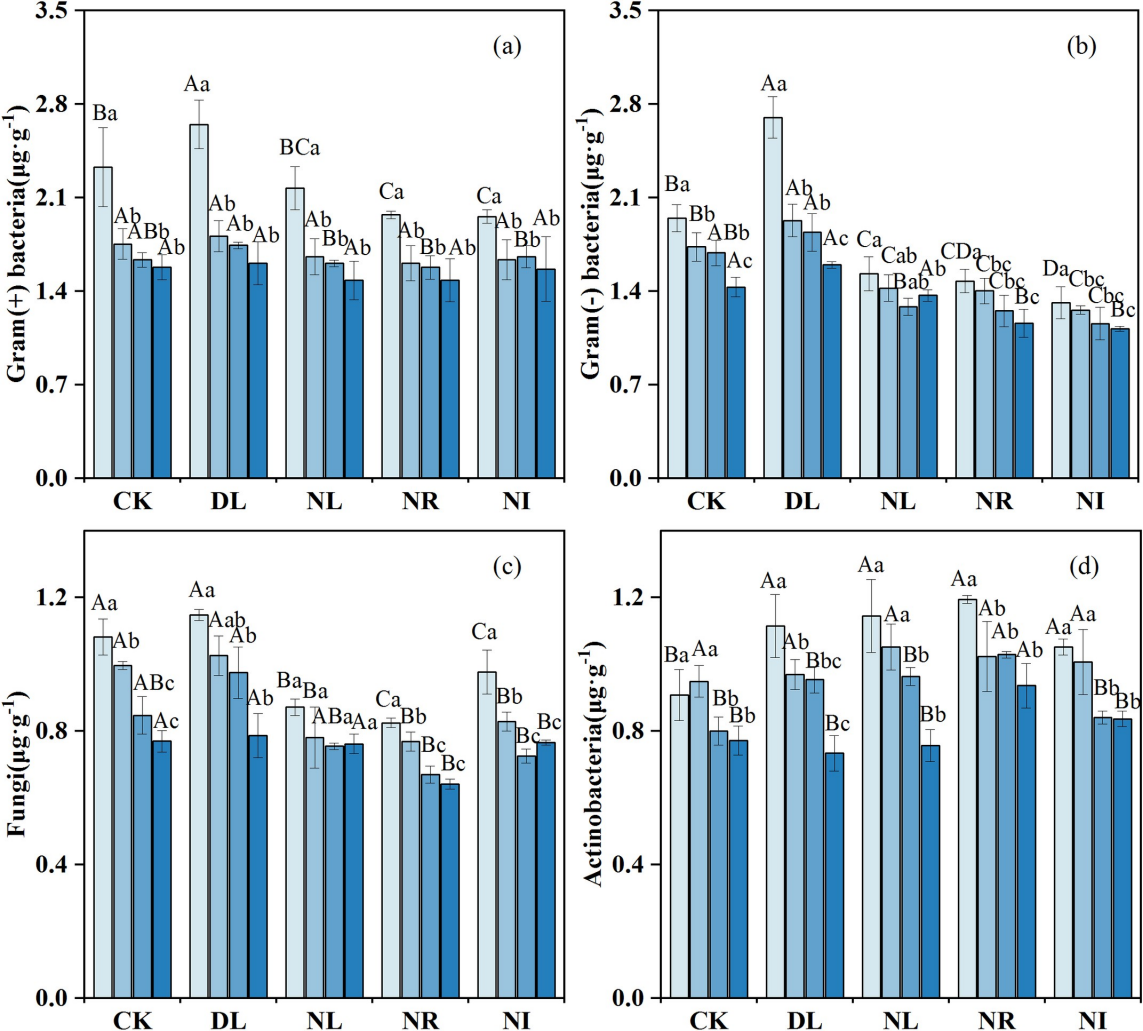
Change in soil PLFA biomass under different treatments (n = 12 for each treatment). CK: control; DL: doubled litter inputs treatment; NL: removal of litter treatment; NR: root removal treatment; NI: root exclusion and litter removal treatment. Lowercase letters indicate differences between soil layers in the same treatment at the *p* < 0.05 level; uppercase letters indicate differences between treatments within the same soil layer at the *p* < 0.05 level.

As shown in Fig 5, litter and root manipulations had different effects on soil microbial biomass. The biomass of gram-positive bacteria, gram-negative bacteria and actinomycetes in the 0–10 cm layer was, 13.68%, 13.14% and 22.81% higher, respectively, in the DL treatment than in the CK (gram-positive bacteria: *P* = 0.048, gram-negative bacteria: *P* = 0.001, actinomycetes: *P* = 0.007). The Biomass of gram-positive bacteria, gram-negative bacteria and fungi was 8.97%, 22.11% and 21.36% lower in the NR treatment than in the CK treatment, and 5.15%, 17.50% and 14.17% lower in the NL treatment than in the CK treatment, respectively. However, compared with the CK, the biomass of actinomycetes increased by 14.21%, 21.99% and 8.96% in the NL, NR, and NI treatments, respectively.

### Relationship between soil C and N and biotic and abiotic factors

The interpretation of the soil C, N and their fractions in the first (RDA1) and second axes (RDA2) was 72.0% and 12.1%, respectively, and the cumulative interpretation of C and N indicators was 84.1% (Table 1). The cumulative interpretation of the relationship between soil C, N and their indicators and other factors reached 88.4%. Therefore, the first two axes can better explain the relationship between soil C, N and soil environmental factors, and it is mainly determined by the first axis.

**Table 1:** RDA of the eigenvalues of soil C and N.

Axes	1	2	3	4
Eigenvalues	72.0%	12.1%	2.9%	1.6%
Explained variation (cumulative)	71.9%	84.1%	87.0%	88.6%
Cumulative eigenvalues of the relationship between soil carbon and nitrogen and other soil factors	96.8%	88.4%	77.7%	92.38%

**Fig 6:**
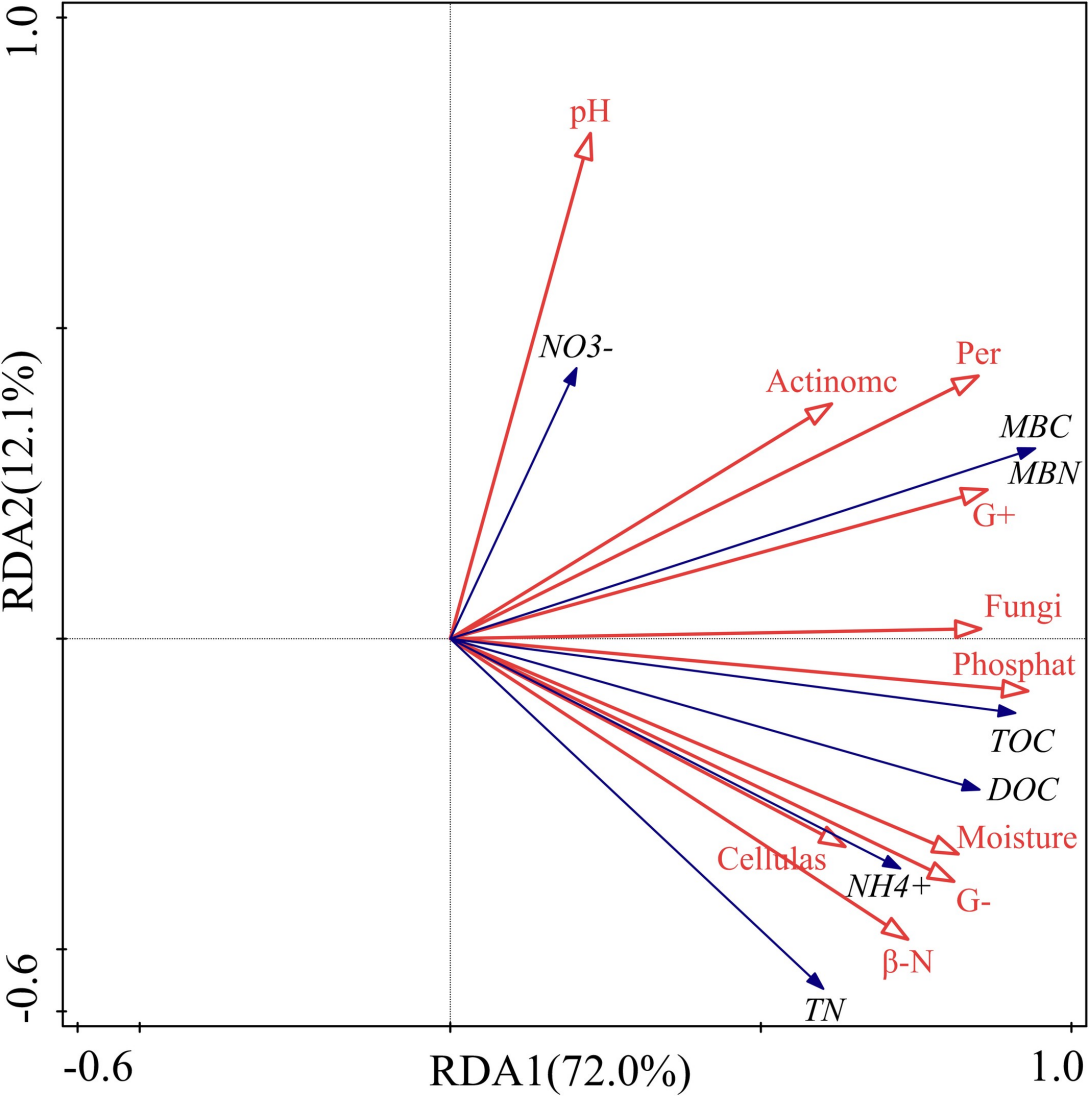
Redundancy analysis of soil carbon and nitrogen, enzyme activity and microorganisms. The filled arrow represents soil carbon and nitrogen and its fractions, and the open arrows represent soil physical, chemical and biotic factors. Symbols: pH, soil pH; NO3-, soil nitrate nitrogen; Actinomc, soil actinomycetes; Per, soil peroxidase; MBC, soil microbial biomass carbon; MBN, soil microbial biomass nitrogen; G+, gram-positive bacteria; Fungi, soil fungi; Phosphate, soil phosphatase; TOC, soil organic carbon; DOC, soil dissolved organic carbon; Moisture, soil moisture content; G-, gram-negative bacteria; NH4+, soil ammonium nitrogen; Cellulase, soil cellulase; β-N, soil β -N-acetylglucosaminidase; TN, total nitrogen.

The RDA indicated that the soil β-N-acetylglucosaminidase activity had a strong positive correlation with soil ammonium N concentration (Fig 6). Likewise, it showed a strong positive correlation between SOC and phosphatase activity. There was also a remarkable positive correlation between DOC and cellulase activity. Except for soil pH, the interpretation of the first seven factors for soil C and N and their fractions were 62.3%, 54.5%, 54.2%, 52.9%, 49.9%, 49.4%, 42.3%, 31.5%, and 29.5% (Table 2). They were important factors affecting soil C and N concentrations under different litter and root manipulations.

**Table 2:** Importance ranking and significance test of physical and chemical factor explanatory quantity.

Index	Order of importance	Interpretation of environmental factors /%	*F*	*P*
phosphatase	1	62.3	29.8	0.002
G+	2	54.5	21.6	0.002
per	3	54.2	21.3	0.002
Fungi	4	52.9	20.2	0.002
Moisture	5	49.9	17.9	0.002
G-	6	49.4	17.6	0.002
β-N	7	42.3	13.2	0.002
cellulase	8	31.5	8.3	0.006
Actinomycetes	9	29.5	7.5	0.008
pH	10	11.8	2.4	0.11

Symbols: pH, soil pH; Actinomc, soil actinomycetes; Per, soil peroxidase; G^+^, gram-positive bacteria; Fungi, soil fungi; Phosphate, soil phosphatase; Moisture, soil moisture content; G-, gram-negative bacteria; cellulase, soil cellulase; β-N, soil β—N-acetylglucosaminidase.

## Discussion

### Effects of litter addition on soil C, N, enzyme activity and microorganisms

Litter is an important link between the material cycle and energy flow in forests, providing nutrients for soil through microbial decomposition and leaching, thereby affecting soil C and N recycling [49, 50]. Our results showed that DL treatment has no significant effect on SOC concentration but significantly increases soil DOC concentration. Previous studies have demonstrated that adding fresh litter can increase the amount of activated C input into the soil and induce the decomposition of old SOC [20, 51]. Increasing low-quality litter can cause indigenous soil microorganisms to grow vigorously and accelerate the decomposition rate of pre-existing soil organic matter, and the excess C will return to the atmosphere in the form of CO_2_ [52]. Thus, the decomposition of pre-existing soil C offsets the new C input into the soil by litter [20]. Moreover, changes in SOC can take a long time to be detected; therefore, the 2-years is likely not sufficient to detect significant changes. This finding is inconsistent with the results by Cusack et al. (2018) [12] that the litter can significantly increase SOC, presumably due to the differences in tree species, litter quality and soil C saturation. DOC and MBC were more sensitive to litter addition, likely because litter addition increases the concentration of available organic matter in soil [53]. Another potential explanation for our results is that doubling the litter may provide a favorable microenvironment and metabolism materials for soil microorganisms, improve microbial biomass, and increase soil DOC concentrations [4, 54]. Furthermore, lignin degradation can also increase the production of soil DOC. Phenols produced by lignin degradation under the DL treatment increased the source of soil DOC [55].

Soil N is a crucial element for plant growth, while plant debris, in turn, can affect the dynamics and transformation of forest soil N. In this study, the DL treatment had no significant on soil TN but led to a marked increase in the soil MBN, ammonium N and nitrate N concentrations. Doubling the litter alters the decomposition rate of litter, provides numerous soluble substances and carbohydrates to the soil and increases soil N input [16, 56]. Additionally, the increase in litter improves the soil hydrothermal environment and increases the biomass of bacteria and fungi in the litter layer and the rate of soil N mineralization, eventually triggering increases in soil MBN and inorganic N [57, 58]. Our results do not conform with the results of Rinnan et al. (2008) [59], who found that litter addition had no remarkable effect on soil ammonium N and MBN in a subarctic heath ecosystem. This difference presumably was a result of the difference in microbial processes caused by climate conditions and experiment durations.

Soil enzymes and microorganisms are involved in the soil nutrient cycle, energy flow and organic matter decomposition and transformation, which are closely related to soil C and N dynamics [60]. In addition to β-N-acetylglucosaminidase, doubling the litter significantly increased soil cellulase, phosphatase, peroxidase activity and soil microbial biomass. Given that litter addition improves soil water, gas, heat and other factors and increases soil C and N concentrations, it would also provide additional substrate sources for soil enzymes and microorganisms and promote the growth and metabolism of soil microorganisms [4, 7]. Another possible explanation for this phenomenon is that litter will also release enzymes to the soil during the decomposition process [61]. However, for β-N-acetylglucosaminidase, DL can increase the available N in the soil and provide the N content required for the growth of microorganisms and plants. Soil microorganisms do not need to secrete a large amount of β-N-acetylglucosaminidase to obtain N, so it does not increase significantly [62].

### Effects of combined root exclusion and litter removal on soil C, N, enzyme activity, and microorganisms

In this study, soil C and N concentrations decreased in the NL and NR treatments. We attribute this finding to two mechanisms. First, the removal of plant detritus blocks exudates from litter and root systems and reduces the input of soil active C and N [63]. Second, the removal of litter and roots reduced the soil water retention capacity and accelerated the leaching of soil nutrients [64], which resulted in a decrease in soil C and N concentrations. Similarly, our observations of decreased soil microbial biomass (gram-positive bacteria, gram-negative bacteria and fungi) in the NL and NR treatments are presumably driven by the decline in soil nutrients and changes in the soil microbial habitat. The removal of litter and root reduces the availability of soil nutrients and the substrate C source required for microbial activity [65]. Imbalanced soil nutrients can inhibit microbial activity. Alternatively, the soil is exposed to intense light and rain when the litter is removed [66], creating suboptimal or lethal conditions for soil microorganisms, possibly damaging the mycelium structure [67]. Given that soil microorganisms are closely associated with the production of enzymes [68], the soil enzyme activity is correspondingly reduced with the decrease in soil microorganisms. Furthermore, removing plant detritus reduces soil enzyme activity by lowering the substrate concentration of the soil enzymatic reaction [69].

We found that the soil nitrate concentration increased in the NL and NR treatments. We speculate that removing litter increases the contact between the soil and atmosphere, improving soil ventilation, and providing sufficient oxygen for soil nitrification [65]. Moreover, the exclusion of roots relieved the inhibition of phenols and organic acids on soil nitrification and blocked soil nitrates removal by nearby plants [70, 71]. The combination of the two increased the soil nitrate N concentration.

Our results revealed that the NL and NR treatments increased soil actinomycete biomass relative to the CK. This result may be because NL reduces the soil water content, alleviates the competition between soil actinomycetes and other bacterial groups, and thus increases the number of drought-resistant actinomycetes [72]. In addition, previous studies have indicated that soil actinomycetes are associated with the degradation of refractory C, such as lignin, and thus actinomycete numbers can increase as the number of live roots decreases [73]. Our results also revealed that NR had a greater impact on soil C and N characteristics and biological activities than the NL, congruent with previous findings [11, 63]. As the main source of organic matter, roots, are in direct contact with mineral soil [74], and litter must input nutrients into the soil through leaching and decomposition [75, 76]. NR reduced root exudates and mycorrhizal hyphae and inhibited the formation of soil aggregates [77], thereby reducing the physical protection of SOC stability [78]. Furthermore, the accumulation of root-derived aliphatic compounds (a source of organic carbon) in soil was greater than that in litter [79]. Another possible explanation is the slow decomposition rate of needles and the short experiment duration relative to the average residence time of litter [80].

### Relationship between soil C and N and other factors under different litter inputs

Changes in litter quantity and quality can affect soil C and N dynamics by affecting the soil environment and biological activity [81, 82]. Several studies have demonstrated that microorganisms are the crucial driving factors for cycling soil C and N [83]. We found that soil microbial groups were positively correlated with soil C and N concentrations. This finding may be because the change in the amount of plant detritus under different treatments affects the C and N required for microbial metabolism, altering the rate of microbial decomposition of organic matter, humus synthesis, and C mineralization [9, 84]. Additionally, microorganisms store soil C and other elements in their cells. Therefore, soil microorganisms are closely related to changes in soil nutrients.

Soil enzymes are involved in soil C mineralization, oxidation-reduction and other processes, which are the main driving factors of soil nutrient cycling [85]. In this study, phosphatase activity is closely related to SOC. It seems likely that phosphatase changes the content of soil nutrients by enzymatic reactions, participates in the decomposition and mineralization of SOC, and promotes the transformation of soil C and N [86]. A remarkable positive correlation between DOC and cellulase was observed in this study, suggesting that the soil cellulase decomposes insoluble cellulose and lignin into water-soluble cellobiose, fructose and other small molecules, promoting the formation of active organic carbon [87]. Conversely, the increase in active organic C will provide sufficient substrate sources for enzymatic reactions. We found a remarkable positive correlation between β-N-acetylglucosidase and soil ammonium N concentration. It may be that β- N-acetylglucosidase can transform chitin into amino sugars. Amino sugars are crucial components and sources of soil active organic N and mineral N [88].

## Conclusions

This study demonstrates that detritus manipulations have different effects on soil properties over a short, two-year period. DL treatment significantly increased soil active C, inorganic N, microbial biomass and enzymatic activity in the surface soil. Litter and root removal significantly reduced soil C, N, enzyme activity and microbial biomass. Collectively, the effect of the belowground litter on soil C, N and biological characteristics was greater than that of the aboveground litter. We found that the above- and belowground detritus input controlled the alteration of soil C and N by changing biotic (enzyme activity and microbial biomass) and abiotic (water content) factors in the studied Schrenk’s spruce forest. These results are of great significance for understanding of the soil C and N turnover of Schrenk’s spruce forest under global change. Future studies are to increase molecular and microbial levels understanding and keep observing over a longer time period, which will help improve understanding of forest soil C and N stability turnover mechanisms.

## Supporting information

S1 Data (ZIP)
